# Mindfulness-Oriented Meditation for Primary School Children: Effects on Attention and Psychological Well-Being

**DOI:** 10.3389/fpsyg.2016.00805

**Published:** 2016-06-07

**Authors:** Cristiano Crescentini, Viviana Capurso, Samantha Furlan, Franco Fabbro

**Affiliations:** ^1^Department of Human Sciences, University of UdineUdine, Italy; ^2^Department of Psychology, Sapienza University of RomeRome, Italy; ^3^Degree Course in Education Science, University of UdineUdine, Italy; ^4^PERCRO Perceptual Robotics Laboratory, Scuola Superiore Sant’AnnaPisa, Italy

**Keywords:** primary school children, mindfulness-meditation, teachers’ report, attention, psychological well-being

## Abstract

Mindfulness-based interventions are increasingly being used as methods to promote psychological well-being of clinical and non-clinical adult populations. Much less is known, however, on the feasibility of these forms of mental training on healthy primary school students. Here, we tested the effects of a mindfulness-meditation training on a group of 16 healthy children within 7–8 years of age from an Italian primary school. An active control condition focused on emotion awareness was employed on a group of 15 age-matched healthy children from the same school. Both programs were delivered by the same instructors three times per week, for 8 total weeks. The same main teacher of the two classes did not participate in the trainings but she completed questionnaires aimed at giving comprehensive pre-post training evaluations of behavior, social, emotion, and attention regulation skills in the children. A children’s self-report measure of mood and depressive symptoms was also used. From the teacher’s reports we found a specific positive effect of the mindfulness-meditation training in reducing attention problems and also positive effects of both trainings in reducing children’s internalizing problems. However, subjectively, no child in either group reported less depressive symptoms after the trainings. The findings were interpreted as suggestive of a positive effect of mindfulness-meditation on several children’s psychological well-being dimensions and were also discussed in light of the discrepancy between teacher and children’s reports. More generally, the results were held to speak in favor of the effectiveness of mindfulness-based interventions for healthy primary school children.

## Introduction

Mindfulness is an attribute of consciousness that can be defined as the ability of paying intentional attention to present moment experience with an open, curious and non-judgmental attitude (Brown and Ryan, 2003; Bishop et al., 2004). A core assumption of mindfulness is that people generally live with an “automatic propensity” that often makes them unaware of their behavioral patterns and of their continuous past- and future-related thoughts and ruminations. This condition of “mindlessness” may contribute to health and psychological problems (e.g., anxiety, depression, emotion dysregulation, and negative mood) (Kabat-Zinn, 1990; Brown and Ryan, 2003; Didonna, 2009; Hölzel et al., 2011). On the contrary, mindful awareness, which can be effectively developed through meditation practice, allows individuals to stay in the here and now and to experience present-moment reality with an open and accepting attitude. This can result in more flexible, adaptive behavior with consequent beneficial health effects at both physical and mental levels (Kabat-Zinn, 1994; Davidson et al., 2003; Didonna, 2009).

Since early eighties onward, mindfulness-meditation techniques have shown to be beneficial in the treatment of different clinical disorders such as chronic pain (Kabat-Zinn, 1982; Brown and Ryan, 2003; Didonna, 2009), eating disorders (Kristeller and Hallett, 1999), anxiety and depression (Miller et al., 1995; Teasdale et al., 2000; Hofmann et al., 2010). In addition to its clinical benefits, a growing body of research focused on testing the cognitive effects that appear to result from this form of cognitive/mental training procedure. Thus, the “observe and accept” approach of mindfulness-meditation has been documented to result in better executive functioning and attention regulation abilities (Jha et al., 2007; Malinowski, 2013).

So far, most of the research on mindfulness-meditation has focused on adults, while only recently the interest on children and adolescents has grown (Zoogman et al., 2014). Preliminary studies in this newborn field suggest that mindfulness-meditation trainings have positive effects on children’s and adolescents’ psychological well-being (Biegel et al., 2009; Burke, 2010; Flook et al., 2010; Semple et al., 2010). However, the status of the overall research is still meager especially in the 1st years of primary school and in healthy children (Zoogman et al., 2014).

To date the restricted body of research examining the effects of mindfulness-meditation trainings on adolescents has reported beneficial effects of this practice for pain management (Thompson and Gauntlett-Gilbert, 2008), depressive relapse prevention (Allen, 2006), reduction of anxiety and depressive symptoms (Beauchemin et al., 2008; Biegel et al., 2009; Broderick and Metz, 2009), and in reducing Attention-Deficit/Hyperactivity Disorder (ADHD) symptoms (Zylowska et al., 2008). Similarly, mindfulness-meditation trainings delivered to children were shown to be useful for reducing anxiety symptoms (Semple et al., 2005; Lee et al., 2008), increasing self-compassion and mindfulness skills (Saltzman and Goldin, 2008), improving social behavior, social skills and attention (Napoli et al., 2005; Saltzman and Goldin, 2008; Semple et al., 2010), reducing ADHD symptoms (Singh et al., 2010), and for improving behavioral regulation, metacognition, and executive functions (Flook et al., 2010). Of importance, if one considers that problems in executive functions are connected with cognitive deficits and with many behavioral disorders like ADHD, as well as with bullying and delinquency (Hughes et al., 2000; Brocki and Bohlin, 2006), then the latter study by Flook et al. (2010) appears particularly relevant. A positive relation was found in this study between improved executive functions observed in children after meditation practice and children’s behavioral regulation, global executive control and metacognition. Thus, mindfulness-meditation would promote enhanced focus and concentration that can then reverberate on children’s behavioral regulation, socio-emotional development, and academic skills (Napoli et al., 2005; Beauchemin et al., 2008; Flook et al., 2010).

In addition to studies on mindfulness-meditation interventions in school-age children, it is worth noting the existence of recent proposals that aim at incorporating mindfulness elements with social-emotional learning (SEL) curriculum into school psychology practice (Felver et al., 2013). On this view, it is suggested that integrating mindfulness into existing schoolwide SEL programs could enhance the effectiveness of the whole intervention with respect to critical children’s social and emotional skills. Moreover, it could help students to maintain focus and clarity and be particularly effective in preventing behavior problems (Felver et al., 2013).

Although extremely valuable, the above mentioned studies on mindfulness-meditation interventions in children have a series of limitations. The majority of these studies have employed no control group (Lee et al., 2008), waiting-list control group (Saltzman and Goldin, 2008) or silent reading control group (Napoli et al., 2005; Flook et al., 2010), rather than active control groups. Furthermore, in these studies children were generally supposed to meditate together with teachers (Napoli et al., 2005) and/or with parents (Saltzman and Goldin, 2008; Singh et al., 2010) and the evaluation of mindfulness-meditation effects on children’s health and behavior was mainly based on parents’ and teachers’ reports. The possible risk could be that they exaggerated the observed changes just because they also had participated in the meditation course (i.e., inflated/placebo effect). In this respect, to the best of our knowledge, there are no previous studies in which parents’ and teachers’ reports (compiled before and after having meditated or not with children) have been compared with children’s self-reported measures. Finally, most of previous mindfulness-based interventions with young participants have been conducted with middle and high school students. Indeed, only a few studies involved younger children at the 1st years of primary school (see in Burke, 2010; Zoogman et al., 2014). Moreover, among these few studies, one was conducted on a restricted clinical sample of 5 anxious children (7–8 years) with no control group (Semple et al., 2005), while the other two studies were conducted on non-clinical samples but did not involve active control conditions (Napoli et al., 2005; Flook et al., 2010).

Therefore, the aim of the current research was to address these problems by investigating in healthy primary school Italian children (7–8 years of age) the health effects of a mindfulness-oriented meditation (MOM) program compared with an active control condition focused on emotion awareness but not involving meditation exercises. In particular, we focused on outcome measures (i.e., the Child Behavior Checklist and the Conners Rating Scales) aimed at giving comprehensive evaluations of several children’s psychological dimensions such as health, cognitive, emotional, social and behavioral processes. While the first outcome measure (i.e., CBCL) is able to distinguish two higher order factors of behavioral problems, namely internalizing and externalizing, the second measure (i.e., the Conners Rating Scales) is particularly appropriate to provide a thorough assessment of attentional functions and related disorders (i.e., ADHD). Of importance, in the current study teachers and parents were not asked to meditate with children and reports from the main teacher of children were collected pre-post trainings together with a children’s self-report measure of mood and depressive symptoms. On the basis of the previous body of research mentioned above, we predicted to find a specific positive effect of MOM versus the control training on attentional functions. Moreover, we expected to find better or similar effects of MOM versus control trainings with respect to other children’s psychological well-being dimensions, such as those measured by both internalizing and externalizing symptoms and behaviors.

## Materials and Methods

### Participants and Setting

Thirty-one children from 2 s year classes of a primary school in the northeast part of Italy (Brugnera, PN) were enrolled in the study. The MOM group was formed by 16 children (eight boys, eight girls) with an age range of 7–8 years (*M* = 7.3, *SD* = 0.5). The control group was formed by 15 children (seven boys, eight girls) also with an age range of 7–8 years (*M* = 7.4, *SD* = 0.5). MOM and control participants were thus matched for age, gender, and education; moreover, children in the two groups had the same ethnic and linguistic background, with every child being Italian mother-tongue. To be included in the study the children had to attend the meetings of the MOM and control trainings for at least six of the eight total weeks (there were three meetings per week). In the MOM group, one child had 3 days of absence, three children had 2 days of absence and two children had 1 day of absence. In the control group, one child had 3 days of absence, one child had 2 days of absence and three children had 1 day of absence. No child was thus excluded from the study.

The two classes were randomly assigned to MOM or active control. Thus, random assignment occurred at the classroom level in the current study. Children’s parents were informed about the activities during a meeting that took place in the school about 1 month before the beginning of the courses. All parents gave consent for their children to participate in the study so that recruitment rate was 100% of eligible students. Children of the two groups received no incentive for participation; moreover, they were blind about the study purpose and did not know they were assigned to a specific group. The children only knew they were expected to work with two trainers on some “exercises” (MOM group) or on reading and commenting a book (control group). Teachers of the two classes were also blind to specific study purpose, specific trainings’ activities and to expected results. The current research followed ethical guidelines and was approved by the Institutional School Board of the “Istituto Comprensivo” di Brugnera, in agreement with the University of Udine. The main teacher of the two classes (having 11 h per week in each class) completed the experimental questionnaires (see next section) approximately 5 days before (baseline session) and 8 days after the completion of the two trainings. For each student, the teacher was required to complete one version of each questionnaire. No teacher was in the classroom during MOM or control trainings. The main teacher just introduced the two instructors (who were the same for the MOM and control trainings) to the classes before the first meeting of the two trainings. The trainings took place in the two classes during school hours. In the same days of the week, children of one class undertook the MOM training and the other class undertook the control training. Both trainings were delivered by the same instructors. The order of MOM and control trainings was randomized.

### Assessment Measures

In this study, we employed two reports from the main teacher and one children’s self-report measure. Each measure was collected to compute possible changes in children’s psychological well-being due to participation in MOM or control trainings. Two different teacher’s paper and pencil measures were used: the Italian versions of the Child Behavior Checklist-Teacher Report Form (CBCL-TRF; Achenbach and Dumenci, 2001; Achenbach and Rescorla, 2001; Italian edition: Frigerio, 2001) and the Conners Teachers Rating Scales – Revised (CTRS-R; Conners, 1997; Italian edition: Nobile et al., 2012). As already mentioned in the Introduction, while there is some overlap in outcomes assessed by the two measures, their combined use appears important to get a better picture of the effects on children’s psychological health caused by the MOM and control trainings. On the one hand, the CBCL-TRF is especially focused on emotional, social and behavioral problems, distinguishing between internalizing and externalizing problem scores within the subject. On the other hand, the CTRS-R is more focused on inattention and ADHD symptoms and is broadly used for the clinical assessment of childhood attentional problems. Moreover, in the context of mindfulness-meditation studies in children, the sole use of the CBCL to test the effectiveness of mindfulness interventions on attentional problems was considered not sufficient (Semple et al., 2010).

The CBCL (Achenbach, 1991; Achenbach and Dumenci, 2001; Achenbach and Rescorla, 2001) is a well-standardized inventory with good reliability and validity (Rescorla et al., 2007; see also Berubé and Achenbach, 2010 and Ang et al., 2012 bibliography). The CBCL-TRF consists of 113 problem-behavior items providing subscores for eight specific problem scales: Anxiety/Depression, Withdrawal/Depression, Somatic Complaints, Social Problems, Thought Problems, Attention Problems, Rule-Breaking Behavior, and Aggressive Behavior. The CBCL-TRF also provides scores for the Total Problems Scale, the Internalizing Problems Scale (expressing an overall *T*-score calculated from the sum of the raw scores of the first three specific problem scales mentioned above), and the Externalizing Problems Scale (giving an overall *T*-score calculated from the sum of the raw scores of the last two specific problem scales). Raw scores for each scale were converted into *T*-scores (*M* = 50, *SD* = 10), based on an original standardization sample of 2,368 children between the ages of 6 and 18 years (Achenbach and Dumenci, 2001; Achenbach and Rescorla, 2001). The teacher rated each child’s behavior on a 3-point scale: 0-Not true (as far as you know), 1-Somewhat or sometimes true, 2-Very true or often true. Scale scores were converted to *T*-scores using age and gender-based norms.

The CTRS-R scale is a 59-item scale with good reliability and validity (Conners et al., 1998; see also Conners, 1997; Nobile et al., 2012). The teacher form of the scale is appropriate for children from 6 to 18 years of age. The CTRS-R measures six types of problems/behaviors: Oppositional, Cognitive Problems/Inattention, Hyperactivity, Anxious-Shy, Perfectionism, and Social Problems. Moreover, the scale has comprehensive symptom coverage for attention deficit/hyperactivity disorder (ADHD), a restless/impulsive scale, an emotional lability scale as well as a “DSM-IV: Inattention” score and a “DSM-IV: Hyperactivity” score (Diagnostic and Statistical Manual of Mental Disorders 4th Edition; American Psychiatric Association [APA], 2000). As for the CBCL-TRF, raw scores from the items of the CTRS-R were converted for each scale into *T*-scores, using age and gender-based norms. *T*-scores are standardized scores with a mean of 50 and a standard deviation of 10. Responses to statements are Likert-type (0 = not true at all, 3 = very much true). Original scale standardization was based on data from teachers of 1,973 children aged 3–17 (Conners, 1997).

A short questionnaire was used as children’s self-report measure: the Short Mood and Feelings Questionnaire (SMFQ, Angold et al., 1995), child version (age 7–16). In particular, the SMFQ is a 13-item scale whose questions are based on the DSM-III criteria for depression and it measures a unidimensional construct of depressive symptoms (Sharp et al., 2006). Responses are Likert-type (0 = not true at all, 2 = very much true) and total score is obtained by summing each item, with a range from 0 to 26, with higher scores denoting higher depressive symptoms (Angold et al., 1995).

### Procedures of the Mindfulness-Oriented Meditation and Active Control Trainings

The MOM training consisted of an 8-week intervention conducted by two mindfulness-meditation instructors with several years of experience with this technique and with education settings (VC and SF). The training was inspired by previous 8-week MOM interventions for clinical and non-clinical adult populations (Fabbro and Muratori, 2012; Campanella et al., 2014; Crescentini et al., 2014, 2015), which were in turn based on the Mindfulness Based Stress Reduction protocol (MBSR; e.g., Kabat-Zinn, 1990, 2003).

The current MOM training was specifically adapted for children and consisted of three meetings per week for a total of 8 weeks. In line with previous mindfulness-meditation programs for healthy children (e.g., Flook et al., 2010), an important characteristic of the MOM training was that meditation periods gradually increased over the 8 weeks (see **Table 1** for an overview of the MOM and control trainings with a brief description of the activities included in each meeting). For the first 2 weeks, the MOM training lasted approximately 30 min per week (i.e., 10 min for each meeting). During week 3 and week 4 it lasted about 45–55 min and it reached a duration of 1 h and a half at the end of the course (week 8). The reason for such adaptations of original MOM trainings (which may include meditation sessions of more than 30 min since the first meeting) was the still immature attentional capacity of children aged 7 or 8 years and their difficulty to engage in a single activity for long periods of time (Posner and Petersen, 1990; Siegler, 1991; see also Semple et al., 2010).

**Table 1:** Overview of the activities included in the MOM and active control training conditions.

Week	Timing per day (min)	Timing per week (min)	Mindfulness-oriented meditation (MOM)	Active control condition
1	9	27	• Feeling the abdomen moving while breathing.	Chapter 1–2
			• Listening to the sounds of the body after a run.	Following the protagonist’s story, try to listen to the heart and try to feel the emotions laying inside.
			• Observing thoughts as they were clouds in the sky.	
2	12	36	• Feeling the hand on the abdomen while breathing.	Chapter 3–4–5
			• Slowly walking in the class feeling every single part of the leg moving.	Try to associate the feelings with the pixies described in the book.
			• Trying to see the main thought – the main cloud - in the mind and writing it down on paper.	
3	15	45	• Feeling a mate’s breath putting the hands on his/her abdomen.	Chapter 6–7
			• Taking a mate’s hand and feeling the contact.	Try to find the pixies that lay in our heart in the different situations.
			• Trying to feel the emotions related to the main thought of the moment.	
4	18	54	• Trying to feel the breath in the nose, without controlling it.	Chapter 8–9
			• Touching the different parts of the face.	Express the feelings related to the emotions laying in the heart.
			• Trying to see the path of a thought: where it comes from and how it disappears.	
5	21	63	• Trying to think the word “in” when air enters the nose, “out” when air comes out from the nose.	Chapter 10–11–12
			• Imaging an object and drawing it. Then observing the chosen object in details and drawing it again.	Try to draw the emotions of the moment.
			• Drawing a mate or a relative and mentally addressing some friendly wishes to the mate (peace, happiness, health).	
6	24	72	• Trying to feel the difference of breath by putting the hands on the throat, on the chest, on the abdomen.	Chapter 13–14–15
			• Raisin meditation: trying to smell, watch, and touch the raisin before eating it.	Try to draw where the emotion found in the heart comes from.
			• Silently watching the eyes of the mates. Trying to understand their thoughts.	
7	27	81	• Feeling the points that air touches when it comes in and out.	Chapter 16–17–18
			• Imaging to be an animal and moving like it.	Try to draw the path of the emotions: where they come from and where they go, if they disappear.
			• Visualizing thoughts as a masquerade parade. Observing them and noticing that they are external to the person.	
8	30	90	• Imaging a little man in the nose that moves, following the air coming in and out.	Chapter 19–20–21
			• Laying down pretending to be a paper and imaging to scan the body, as if you were in a copying machine.	Try to find a still and quiet place in the heart to enter when feeling overwhelmed by emotions.
			• Visualizing thoughts as a masquerade parade, drawing them and observing the differences between thoughts and drawing.	


*A brief description of the activities is given for each week. Activities remained constant for the three meetings of a given week. Chapter in the active control condition refers to chapters of the *Six pixies in my heart* book (Corallo, 2011).*

Each meeting was divided into a series of three meditation exercises, which focused on three types of activities: (i) mindfulness of breathing, (ii) mindfulness of body parts, (iii) and mindfulness of thoughts. In more details, the three meditation activities were proposed to children as exercises or “games” that were meant to promote awareness of the three aspects of the self, related to breath, body parts and thoughts. In each of the 3 weekly meetings children were first required to concentrate on breath refraining from actively controlling it. In the second meditation exercise they had to kindly focus their attention on different body parts. In the last activity children were encouraged to observe the stream of their thoughts and emotions. Children were supported in carrying out these exercises through the use of tools or mental images. For example a pencil case on the belly was used to better focus and observe the breath, while imaging thoughts as soap bubbles, sea waves or clouds was believed to help children experiencing and understanding the transitory process of thoughts. During the body contemplation exercises, we also used meditation in movement whereby children were asked to mindfully explore their body while they were walking imaging that the floor was made of sand or grass. In each meditation activity, children were encouraged to gently draw attention back to the task without judging themselves when they noticed that their attention was wandering. After each meditation exercise there was a debriefing phase in which trainers explained the next exercise and children could express their feelings and questions about the exercise just completed. Globally, the debriefing phase lasted approximately half the phase dedicated to meditation exercises.

The activities of the control group were designed to be comparable and structurally equivalent to those of the MOM training (see MacCoon et al., 2012 for a discussion and proposal of active control interventions in mindfulness-meditation studies on adults). Thus, similarly to the MOM course, the control training was also organized in a group format of a series of three meetings per week for 8 total weeks. Control participants completed the same amount of class practice as the children in the MOM group.

The activities of the control group consisted in reading and commenting the different chapters of the book: *Six pixies in my heart* (“Sei folletti nel mio cuore”, Corallo, 2011). The book is divided into 21 chapters, so that, based on the number of pages, two or three chapters were presented to children each week. *Six pixies in my heart* is about a shy and sensitive child deciding to start a path to avoid all his emotions with the aim of not being defined “sensitive” from his friends and school mates. However, at the end of the book, the child learns the importance of feeling positive and negative emotions in his heart and appreciates the fact of being sensitive (see **Table 1** for a brief description of the activities included in the control training).

Similarly to the organization of meetings in the MOM group, each meeting for the control group was divided in a reading part and a discussion part. Duration of reading and commenting parts followed the same progression used in the MOM course: they gradually increased over the 8-week period, starting from 30 min per week during week 1 to reach 1 h and a half per week at the end of the course. The activities of listening and commenting the stories reported in the chapters allowed children to discover all the different emotions and feelings that can be experienced in different situations. This was an indirect training on emotion awareness and acceptance since it implicitly encouraged children to consider their own emotions. In the MOM group instead, children were explicitly asked to focus attention on breath, mind, and body in order to observe and accept any arising feeling, emotion, and thought. In sum, the control training shared several crucial elements with the MOM training, including specific active ingredients (MacCoon et al., 2012) meant to enhance psychological well-being of children but designed to be non-specifically related to the practice of mindfulness. These active ingredients were timing and setting, the group work, the interaction between students and trainers, and the conditions of silence and concentration that were required to children. It should be noted that silent reading/listening (without comment and discussion) has often been used for adults and children as control training condition, or as an activity included in other active control trainings (MacCoon et al., 2012), in mindfulness-meditation studies (Napoli et al., 2005; Flook et al., 2010; Zeidan et al., 2010). Of importance, an effect of silent reading/listening in enhancing participants’ mood but not executive function was found in these previous studies. To conclude, the present control training appeared appropriate as an active control condition for MOM research in children.

### Statistical Methods

The data were analyzed with Statistica 8 (StatSoft, Inc, Tulsa, OK). *T*-scores were used for the CBCL-TRF and the CTRS-R scales and subscales while raw scores were used for the children’s self-report measure (SMFQ). For the CTRS-R and CBCL-TRF measures, separate multivariate analyses of variance (MANOVAs) were performed to determine whether children from the MOM group differed significantly in their behaviors from children from the control group. More specifically, for the CBCL-TRF measure we focused on the two broadband factors of internalizing and externalizing behavioral problems as well as on the scores of total behavioral problems (see McFarlane et al., 2003 for a similar approach to CBCL data). The analysis included Group (MOM, controls) as between-subject factor and the pre-post trainings internalizing, externalizing, and total behavior problems scales as dependent variables. For the CTRS-R scale, the MANOVA included Group as between-subject factor and, as dependent variables, it specifically focused on the factors that most directly map onto the ADHD and oppositional spectrum of behaviors in the DSM-IV (see Purpura and Lonigan, 2009 for a similar approach to the CTRS scale). The analysis included the data from the following scales: oppositional behaviors, cognitive problems/inattention, hyperactivity, ADHD index, CGI restless/impulsive behaviors, DSM-IV: Inattention, and DSM-IV: Hyperactivity. In all analyses, significant main-effects and interactions were followed-up with univariate pair-wise comparisons (with Bonferroni correction for multiple comparisons applied). Finally, for the SMFQ data we ran a mixed model analysis of variance (ANOVA) involving the within-subject factor of TIME (pre-training, post-training) and the between-subject factor of Group (MOM, controls). The significance threshold of *p* < 0.05 was used in all statistical tests. In the analyses, effect sizes are reported as $\eta_{p}^{2}$.

## Results

### Teacher’s Report: Child Behavior Checklist-Teacher Report Form

*T*-scores for both groups of children are shown in **Table 2** for each problem scale and subscale of the CBCL-TRF (i.e., total problems, internalizing problems and externalizing problems scales considered in the following MANOVA, and the eight specific problem scales). At both testing sessions (i.e., before and after the trainings), children’s mean *T*-scores appeared to be in the normal range. In the CBCL-TRF, *T*-scores less than 67 and less than 60 are indeed considered in the normal range, respectively, for the eight syndrome scales and for the total problems, externalizing problems, and internalizing problems scales (Achenbach and Dumenci, 2001; Achenbach and Rescorla, 2001).

**Table 2:** Mean *T*-scores and standard deviations (in parentheses) obtained by children in the MOM and control groups in the two testing sessions (i.e., before and after the trainings).

CBCL-TRF	Pre-training MOM group *M T*-score *(SD)*	Post-training MOM group *M T*-score *(SD)*	Pre-training control group *M T*-score *(SD)*	Post-training control group *M T*-score *(SD)*
I. Anxious/Depressed	59.88 (8.53)	59.19 (7.73)	54.53 (4.91)	54.53 (4.51)
II. Withdrawn/Depressed	56.31 (6.21)	55.63 (5.43)	57.33 (6.25)	56.20 (6.31)
III. Somatic complaints	53.13 (4.30)	52.69 (4.23)	51.87 (4.24)	51.60 (4.22)
IV. Social Problems	55.56 (5.81)	54.94 (5.37)	55.13 (5.76)	54.93 (5.56)
V. Thought Problems	52.50 (4.85)	52.50 (4.85)	50.93 (2.46)	50.93 (2.46)
VI. Attention Problems	53.94 (5.01)	52.81 (3.63)	52.27 (3.61)	52.27 (3.61)
VII. Rule-breaking behavior	53.69 (5.51)	52.88 (4.16)	54.33 (4.49)	53.60 (4.53)
VIII. Aggressive behavior	54.06 (4.31)	53.88 (4.09)	55.53 (4.58)	55.27 (4.18)
Internalizing problems^∗∗^ (I+II+III)	57.25 (9.56)	56.38 (9.20)	53.93 (7.08)	53.27 (6.77)
Externalizing problems (VII+VIII)	51.88 (7.32)	51.06 (7.22)	53.80 (6.51)	53.47 (6.23)
Total score^∗^ (IV+V+VI+Internalizing+ Externalizing +Other problems^#^)	53.06 (8.59)	51.75 (7.77)	52.00 (5.58)	51.00 (6.69)


*CBCL-TRF stands for Child Behavior Checklist-Teacher Report Form; ^#^ The Other problems scale refers to 17 items that are not computed as a single scale but are involved in the computation of the Total score; ^∗^ Indicates the scales for which significant effects were found in the MANOVA analysis after the application of a Bonferroni correction for multiple comparisons; ^∗∗^ Indicates the scales for which marginally significant effects were found in the MANOVA analysis after the application of the Bonferroni correction (see main text for further details). Scales I through VIII were not individually analyzed but the data are still reported in the table.*

A MANOVA was performed on internalizing, externalizing, and total behavior problems scores between children from the MOM and the control groups. The analysis included the scores measured both before and after the trainings (i.e., the factor of TIME at two levels: pre- and post-training). Results indicated that there were no significant differences between groups [*F*(3,27) = 0.91, *p* = 0.450; $\eta_{p}^{2}$ = 0.091] and that the Group factor did not interact with the TIME factor [*F*(3,27) = 0.15, *p* = 0.927; $\eta_{p}^{2}$ = 0.016]. However, the main effect of TIME was significant [*F*(3,27) = 3.69, *p* = 0.024; $\eta_{p}^{2}$ = 0.291] indicating lower scores globally on the three scales at post- versus pre-training (**Table 2**). After adjusting α to 0.017 (i.e., a Bonferroni correction of three was applied) to control for an inflated type I error, planned pair-wise comparisons showed that this effect was due in particular to total behavior problems [*F*(1,29) = 10.77, *p* = 0.002] and more marginally to internalizing behaviors [*F*(1,29) = 6.13, *p* = 0.019] [*F*(1,29) = 2.38, *p* = 0.133 for externalizing behaviors]. Thus, the data from the CBCL-TRF highlighted the effectiveness of both types of trainings in reducing total behavior problems and, more marginally, internalizing problems.

### Teacher’s Report: Conners Teacher Rating Scale-Revised (CTRS-R)

As already mentioned, CTRS-R was chosen because of its appropriateness for the study of Children’s attentional functions and related disorders (i.e., ADHD). Accordingly, among the behavior and symptoms covered by the CTRS-R, we focused the analysis specifically on the scales related to attention function/ADHD symptoms. The analysis did not include the scales overlapping to some extent with the types of problems (e.g., internalizing problems) already assessed by the CBCL-TRF (i.e., Anxious-Shy, Perfectionism, Social Problems, CGI-Emotional Lability, and CGI-Total scales). *T*-scores for both groups of children are shown in **Table 3** for each scale and index of the CTRS-R (i.e., the Oppositional, Cognitive Problems/Inattention, Hyperactivity, ADHD index, DSM-IV: Inattention, DSM-IV: Hyperactivity, CGI-Restless/Impulsive scales considered in the following MANOVA and the Anxious-Shy, Perfectionism, Social Problems, CGI-Emotional Lability, and CGI-Total scales). Children’s mean *T*-scores can be classified in the normal range as all of them were less than the threshold value of 60 (Conners, 1997).

**Table 3:** Mean *T*-scores and standard deviations (in parentheses) obtained by children in the MOM and control groups in the two testing sessions (i.e., before and after the trainings).

CTRS – R	Pre-training MOM group *M T*-score *(SD)*	Post-training MOM group *M T*-score *(SD)*	Pre-training control group *M T*-score *(SD)*	Post-training control group *M T*-score *(SD)*
Oppositional	49 (6.76)	48.81 (7.12)	49.46 (5.99)	48.8 (5.11)
Cognitive Problems/Inattention^∗^	49.81 (8.63)	47.75 (5.49)	45.73 (3.01)	45.6 (2.84)
Hyperactivity	48.37 (6.84)	47.87 (6.39)	47.2 (5.33)	47.13 (5.71)
Anxious-Shy	48.56 (9.23)	47.12 (8.13)	44.06 (4.23)	43.26 (3.89)
Perfectionism	45.81 (7.79)	45.12 (7.01)	44.26 (5.67)	43.46 (4.47)
Social Problems	53.12 (9.72)	52 (8.54)	51.06 (5.75)	50 (6.30)
ADHD Index^∗^	48.06 (6.60)	46.37 (4.93)	46.33 (5.15)	46.46 (5.22)
DSM-IV: Inattention^∗∗^	50.68 (10.16)	48.75 (6.64)	46.73 (5.02)	46.53 (5.04)
DSM-IV: Hyperactivity	47.50 (4.85)	47.31 (4.46)	46.93 (5.25)	47 (5.81)
CGI: Restless-Impulsive^∗^	48.25 (6.90)	46.62 (6.14)	45.86 (5.26)	45.73 (5.11)
CGI: Emotional Lability	49.87 (7.75)	48.06 (6.58)	46.13 (5.19)	46 (5.19)
CGI: Total	48.5 (7.08)	46.81 (6.36)	45.66 (5.17)	45.46 (4.88)


*CTRS-R stands for Conners Teacher Rating Scale-Revised; ADHD stands for Attention deficit/hyperactivity disorder; CGI stands for Conners Global Index; DSM-IV stands for Diagnostic and Statistical Manual of Mental Disorders 4th Edition. ^∗^ Indicates the scales for which significant effects were found in the MANOVA analysis after the application of a Bonferroni correction for multiple comparisons; ^∗∗^ Indicates the scales for which marginally significant effects were found in the MANOVA analysis after the application of the Bonferroni correction (see main text for further details). The anxious-shy, perfectionism, social problems, CGI: Emotional Lability, and CGI: Total scores were not individually analyzed but the data are still reported in the table.*

The MANOVA was performed between children from the MOM and control groups on scores obtained in the scales measuring oppositional, cognitive problems/inattention, hyperactivity, ADHD index, CGI restless/impulsive, DSM-IV: Inattention, and DSM-IV: Hyperactivity behaviors. The analysis included the scores measured both before and after the trainings. Results indicated that there were no significant differences between groups [*F*(7,23) = 0.93, *p* = 0.503; $\eta_{p}^{2}$ = 0.220]. The main effect of TIME was also non-significant [*F*(7,23) = 2.07, *p* = 0.089; $\eta_{p}^{2}$ = 0.386]; however, the two-way TIME × Group interaction was significant [*F*(7,23) = 3.12, *p* = 0.018; $\eta_{p}^{2}$ = 0.487]. After adjusting α to 0.007 (i.e., a Bonferroni correction of seven was applied), planned pair-wise comparisons showed no pre-post training significant differences in any of the seven scale scores for children from the control training [all *F*(1,29) < 0.85, *p* > .350). Nevertheless, MOM children showed reduced scores after the training for the following scales: Cognitive Problems/Inattention [*F*(1,29) = 8.63, *p* = 0.006], ADHD index [*F*(1,29) = 16.27, *p* = 0.001], and CGI restless/impulsive *F*(1,29) = 23.80, *p* = 0.001]. There was also a trend for the DSM-IV: Inattention scale [*F*(1,29) = 6.32, *p* = 0.017]. For the remaining three scales (Oppositional, Hyperactivity, and DSM-IV: Hyperactivity) there was no reliable change in the scores due to participation in the MOM course [all *F*(1,29) < 3.76, *p* > 0.061] (**Table 3**).

Overall, the results indicated a specific effect of the MOM training in reducing problems associated with ADHD and in particular those concerning inattention.

### Children’s Self-Report: Short Mood and Feelings Questionnaire

Short Mood and Feelings Questionnaire scores for both groups of children are shown in **Table 4**. One child of the MOM group was absent during compilation of the self-report measure at posttest; this left 15 children in both groups for the SMFQ. The 2 (TIME: pre-training, post-training) × 2 (Group: MOM, controls) ANOVA did not show significant main effects of TIME [*F*(1,28) = 0.94, *p* = 0.338; $\eta_{p}^{2}$ = 0.032) and Group [*F*(1,28) = 0.55, *p* = 0.464; $\eta_{p}^{2}$ = 0.019] or the interaction between these two factors [*F*(1,28) = 0.12, *p* = 0.722; $\eta_{p}^{2}$ = 0.004] (**Table 4**). Thus, no child in either group reported significantly decreased SMFQ scores (i.e., denoting less depressive symptoms) after the trainings.

**Table 4:** Mean and standard deviations (in parentheses) obtained by children in the MOM and control groups in the two testing sessions (i.e., before and after the trainings).

	Pre-training MOM group *M (SD)*	Post-training MOM group *M (SD)*	Pre-training control group *M (SD)*	Post-training control group *M (SD)*
SMFQ	5.60	5.20	6.60	5.73
	(3.98)	(3.12)	(2.97)	(3.21)


*SMFQ stands for Short Mood and Feeling Questionnaire- short version.*

Globally for the teacher’s reports, the data showed specific positive effects of the MOM training in reducing problems associated with ADHD such as inattention and a beneficial effect of both trainings in reducing internalizing and emotional problems. However, subjectively, the children in the MOM or control groups did not report better mood or less depressive symptoms after the trainings.

## Discussion

The aim of the present study was to evaluate the effects of an 8-week MOM training on healthy primary school children. To this end, we used both reports from the children’s main teacher (CBCL-TRF and CTRS-R) and a children’s self-report measure (SMFQ). Moreover, we compared the MOM training with an active control condition. The control group engaged in a work on emotion awareness and recognition, with modalities similar to those used in the MOM training. Based on reports from the teacher, we found specific positive effects of the MOM training in reducing problems associated with ADHD such as inattention (cf. see Teacher’s Report: Conners Teacher Rating Scale-Revised (CTRS-R).). Moreover we also found beneficial effects of both trainings in reducing children’s internalizing problems such as anxiety (cf. see Teacher’s Report: Child Behavior Checklist-Teacher Report Form). However, subjectively, the children in the MOM or control groups did not report better mood or less depressive symptoms after the trainings (cf. see Children’s Self-report: Short Mood and Feelings Questionnaire.).

Overall, the present findings significantly extend prior research on mindfulness-meditation on children’s and adolescents’ psychological health (Napoli et al., 2005; Zylowska et al., 2008; Flook et al., 2010; Semple et al., 2010), by showing positive effects of a MOM training on the attentional skills, ADHD symptoms and emotional functions of a group of healthy primary school children. To the best of our knowledge, the present study is the first to compare, in healthy children of this age, mindfulness-meditation training with a structurally equivalent active control condition focused on emotions awareness and recognition. In particular, the specific experimental design used in the current study allowed us to observe similar positive effects of MOM and active control on children’s total and internalizing problems (i.e., the significant effect of TIME together with the non-significant TIME × Group interaction for the CBCL-TRF), together with the superior effect of MOM for children’s attentional functions (i.e., the significant TIME × Group interaction in the CTRS-R scales measuring attentional skills).

Thus, the current study confirms and extends to primary school children the crucial role of attention in MOM interventions (Zylowska et al., 2008; Flook et al., 2010; see also Malinowski, 2013 for a recent review on mindfulness and attention in adults). It has been argued that self-regulation of attention is a fundamental element of MOM and a prerequisite for the development of other related components. One of these components through which mindfulness-meditation exerts its positive health effects is thought to be emotion regulation (Hölzel et al., 2011; Malinowski, 2013). On this view, the current study may thus indirectly lend support to the idea that mindfulness-meditation facilitates emotion awareness and regulation via increased ability to allocate attentional resources and to monitor the content of one’s own present-moment emotional experience. Following this hypothesis, the children will gain a better ability to notice and accept any arising emotions, decreasing the tendency to overreacting or avoiding them. Nevertheless, while this possibility may account for the positive effects of the MOM training on children’s internalizing and emotional problems, it may not be sufficient to explain the similar effects obtained in the control group. While children in the MOM group had a specific training on attention, the control children made a specific work on emotions awareness and recognition that required them to draw their own conclusions about the importance of emotions in people’s lives. Following the conversational approach to theory of mind (Dunn et al., 1991; Lecce et al., 2014), it is possible that reading and commenting the different chapters of the *Six pixies in my heart* book about the protagonist’s emotions and mental states was sufficient for children to relate these experiences to their own feelings and emotions.

An important aspect of the present study concerns the discrepancy between the main teacher’s reports of reduced internalizing problems observed after both trainings and the absence of any improvement in mood and depressive symptoms as reported by children. A first possibility for this discrepancy is that children may have experienced difficulties in self-reporting due to their not yet fully developed introspective, metacognitive abilities. During meditation, for example, such abilities could favor a detached, positive view of the emotional content of present-moment experience (i.e., detachment or decentering, a fundamental mechanism of mindfulness-meditation, Shapiro et al., 2006; Hölzel et al., 2011). More in particular, it has been argued that, in the earlier stage of the practice, mindfulness-meditation would act as a top-down emotion regulation strategy involving cognitive reappraisal of negative emotions that could not be fully developed and introspectively accessible by children (e.g., McRae et al., 2012; Chiesa et al., 2013). An alternative explanation is to consider teacher’s reports of internalizing symptoms as inherently weaker than children’s reports of the same problems. Besides the fact that individuals are typically more accurate self-reporters of internalizing symptoms than parents or teachers, there is evidence that teachers tend to view internalizing symptoms as less problematic possibly because of their “intropunitive”, rather than overtly or disruptive, character (Tandon et al., 2009). In other words, the teacher’s reports were based on behavioral observations while the children’s report was based on subjective experience of emotions. This difference, especially when interpreted in light of the finding of the effects of the MOM training in reducing ADHD-related problems, may suggest that the MOM training could have been particularly effective in changing behavior problems (other than externalizing disorders) rather than the subjective mood of children in the age group studied. A third possibility can be put forward to explain the lack of change observed in the children’s self-report measure of mood and depressive symptoms (SMFQ). With a range of SMFQ scores being 0–26 and baseline scores for each group being between 5 and 6, the lack of findings could be due to a floor effect for this measure.

Thus, a number of alternative hypotheses can be proposed to explain the discrepancy between teacher’s and children’s reports as well as the specific effects and mechanisms of action of the MOM versus the control trainings. Nonetheless, it should be noted that on the basis of the available data, it is not possible to disentangle these possibilities. Careful comparison of these alternatives awaits future research. Moreover, it should be noted that the present study only focused on mood and feelings when trying to compare the effects of the two trainings from the perspective of the main teacher and from that of the children. Future studies collecting self-report measures of attention are needed to clarify whether the discrepancy between the main teacher’s reports and the children’s reports occurs also for attentional functions.

Overall, the present findings point to the feasibility and utility of interventions based on mindfulness-meditation in educational contexts involving healthy primary school students by showing positive influences of this form of mental training on several dimensions of children’s psychological well-being. Nevertheless, a number of limitations and suggestions for future research also need to be considered. The first limitation of the current research is the restricted sample size, which although being similar or larger to that of many other studies on mindfulness-based interventions on children and adolescents (Burke, 2010; Zoogman et al., 2014), suggests replication and extension of current findings to larger samples. Moreover, a randomized design at the individual student level rather than at the classroom level would allow one to control for a possible confound due to differences between the classrooms.

Other issues pertain to the type of experimental material and type of trainings used. First of all, in light of the number of alternative hypotheses that can be proposed to explain the discrepancy between teacher’s and children’s reports, it is advisable that future studies will try to extend the present findings by comparing teachers’ and/or parents’ reports with other children’s subjective, self-report measures of attention, mindfulness and psychological health changes. For example, available mindfulness scales such as the Child and Adolescent Mindfulness Measure (CAMM, Greco et al., 2011), which is suitable for children aged 6–18 years, could be used and the possible changes in children’s mindfulness skills could be related to measures of psychological well-being and academic achievements. Indeed, it was shown that CAMM scores are positively correlated with quality of life, academic competence, and social skills and negatively correlated with somatic complaints and internalizing and externalizing problems in children (Greco et al., 2011). Physiological measures of stress reduction assessing, for instance, hormones levels, heart rate, or blood pressure could also be used in children both before and after MOM trainings. Moreover, the specific effect of MOM on children’s attentional functions suggests that future studies may support teacher’s reports with other measures of attention directly collected from children, for instance in the forms of self-report measures or more objective computerized tests such as the Attentional Network Test (ANT, Fan et al., 2002; see Rueda et al., 2004 for the child version of the ANT). This test allows evaluating the function of three distinct attentional networks (alerting, orienting, and executive control), which have been shown to be positively affected by mindfulness meditation in adults and adolescents (e.g., Zylowska et al., 2008; Malinowski, 2013). Future studies may also try to combine the ANT with other behavioral measures of attention and executive functions already explored in past studies of mindfulness meditation in children and adolescents such as the Stroop task and the Trail Making Test (Napoli et al., 2005; Zylowska et al., 2008; Van de Weijer-Bergsma et al., 2012).

The positive effects of MOM on attention and ADHD symptoms, as well as on other behavior problems (e.g., internalizing problems), encourage future applications of mindfulness-based therapies in ADHD, and possibly other disorders, in developmental age. In particular ADHD is a complex and multidimensional disorder on which mindfulness-based interventions have positive effects in terms of better attentional functions and reduced impulsivity, stress, anxiety, and depression symptoms (Zylowska et al., 2008; Van de Weijer-Bergsma et al., 2012). The present MOM training already included some key elements (e.g., walking meditation or exercises of wishing well to self and others) taken from these past mindfulness-based interventions in young ADHD individuals, but it could be further adapted for ADHD samples including, for example, parallel mindful parenting training for parents (e.g., Van der Oord et al., 2012), or asking parents to meditate with their children (Zylowska et al., 2008; Van de Weijer-Bergsma et al., 2012). Moreover, children with ADHD could be helped with shorter meditation sessions, with a stronger emphasis on impact of mindful awareness in everyday life, and using didactic visual aids to explain mindful awareness concepts (see Zylowska et al., 2008 for a detailed report of mindfulness-meditation in ADHD).

From another perspective, it may be important for future studies to collect parents’ reports in addition to the teacher’s reports and compare these reports with those from the children. In our research we decided not to include the parents’ reports, only focusing on the teacher’s reports, because we hypothesized that the parents’ reports could be affected if the children shared with them the experience gained during the MOM course. Future studies may overcome this limitation of the present study by collecting reports from both parents and teachers, and directly from the children, and asking different groups of children to meditate (or carrying out the activities of the active control training) only with the instructors or even with their teachers and parents.

With regards to MOM and control trainings, other issues are worth discussing. A general limitation involving both trainings concerns the lack of a follow-up examination. This lack precludes any precise knowledge of the duration of the changes observed in MOM and control children. Another issue pertains to the physical activity and movement component of the MOM intervention that was not included in the active control condition. We believe that this difference did not have any impact on our outcome measures. Movement was indeed a minimal, secondary part of the MOM training and was conceived as a way to enhance awareness of the body rather than as a mere physical exercise. In line with this, recent evidence suggests that the strength of MOM interventions lies more in the acquisition of self-regulatory skills (e.g., attentional control) than in physical movement. For example, when yoga training – integrated with meditation and breath awareness exercises – was compared to physical education programs in school contexts, yoga showed more pronounced positive effects than physical education on several psychosocial well-being measures (e.g., negative affect, anxiety, mood, and mindfulness; Noggle et al., 2012). Despite these findings and the marginal role of the movement component in our MOM training, it is desirable that future studies compare MOM trainings with other active control conditions such as relaxing activities (e.g., muscular relaxation or relaxation with music) or yoga, or with other health enhancement procedures (MacCoon et al., 2012), which can be suitable for children. Yet another issue that could be addressed more systematically by future research concerns the duration of meditation exercises and number and frequency of meditation sessions in children. In line with other studies on the effects of mindfulness meditation in children (e.g., Flook et al., 2010; Semple et al., 2010; Zoogman et al., 2014; see also Harnett and Dawe, 2012), we showed that gradually increasing length of sessions and duration of structured practices (in addition to having more weekly sessions than in a typical MOM program with adults) led to significant positive effects on children’s psychological health. However, the fact that we have systematically assessed the changes only at the end of the course rather than during it, for example once every 2 weeks, does not allow us to easily identify an ideal amount of time for mindfulness practice in our group of children. We believe, however, that the question of the duration of MOM practices and interventions in children is very important; this is an issue that deserves more systematic investigation, in line with what is recently happening with adults (e.g., Carmody and Baer, 2009; see Greenberg and Harris, 2012 and Harnett and Dawe, 2012 for related arguments in children). Finally, the present findings of positive health effects of our active control condition suggest that future studies could integrate mindfulness and other SEL programs into a single, coherent preventive intervention (Felver et al., 2013; see also Introduction).

## Conclusion

The current longitudinal study showed how the introduction of mindfulness-meditation practices in educational settings can be useful to improve children’s cognitive, emotional, and social abilities. This awareness practice could be regularly used during the school year and, combined with other SEL programs, could become a powerful preventive tool and a mean to improve the academic development of students even in the 1st years of school.

## Author Contributions

CC contributed to the conception, design, acquisition, analysis, and interpretation of data. VC contributed to the acquisition, analysis, and interpretation of data. SF contributed to the acquisition and interpretation of data. FF contributed to the conception, design, and interpretation of data. All authors have participated in the research and article preparation and all approved the final article.

## Conflict of Interest Statement

The authors declare that the research was conducted in the absence of any commercial or financial relationships that could be construed as a potential conflict of interest.

The reviewer AR declared a shared affiliation, though no other collaboration, with one of the authors VC to the handling Editor, who ensured that the process nevertheless met the standards of a fair and objective review.
